# SARS-CoV-2 antibody magnitude and detectability are driven by disease severity, timing, and assay

**DOI:** 10.1126/sciadv.abh3409

**Published:** 2021-07-30

**Authors:** Michael J. Peluso, Saki Takahashi, Jill Hakim, J. Daniel Kelly, Leonel Torres, Nikita S. Iyer, Keirstinne Turcios, Owen Janson, Sadie E. Munter, Cassandra Thanh, Joanna Donatelli, Christopher C. Nixon, Rebecca Hoh, Viva Tai, Emily A. Fehrman, Yanel Hernandez, Matthew A. Spinelli, Monica Gandhi, Mary-Ann Palafox, Ana Vallari, Mary A. Rodgers, John Prostko, John Hackett, Lan Trinh, Terri Wrin, Christos J. Petropoulos, Charles Y. Chiu, Philip J. Norris, Clara DiGermanio, Mars Stone, Michael P. Busch, Susanna K. Elledge, Xin X. Zhou, James A. Wells, Albert Shu, Theodore W. Kurtz, John E. Pak, Wesley Wu, Peter D. Burbelo, Jeffrey I. Cohen, Rachel L. Rutishauser, Jeffrey N. Martin, Steven G. Deeks, Timothy J. Henrich, Isabel Rodriguez-Barraquer, Bryan Greenhouse

**Affiliations:** 1 Division of HIV, Infectious Diseases, and Global Medicine, University of California, San Francisco, San Francisco, CA, USA.; 2 Department of Epidemiology and Biostatistics, University of California, San Francisco, San Francisco, CA, USA.; 3 Division of Experimental Medicine, University of California, San Francisco, San Francisco, CA, USA.; 4 Abbott Laboratories, Abbott Park, IL, USA.; 5 Monogram Biosciences Inc., South San Francisco, CA, USA.; 6 Department of Laboratory Medicine, University of California, San Francisco, San Francisco, CA, USA.; 7 Division of Infectious Diseases, University of California, San Francisco, San Francisco, CA, USA.; 8 UCSF-Abbott Viral Diagnostics and Discovery Center, San Francisco, CA, USA.; 9 Vitalant Research Institute, San Francisco, CA, USA.; 10 Department of Pharmaceutical Chemistry, University of California, San Francisco, San Francisco, CA, USA.; 11 Department of Cellular and Molecular Pharmacology, University of California, San Francisco, San Francisco, CA, USA.; 12 Chan Zuckerberg Biohub, San Francisco, CA, USA.; 13 National Institute of Dental and Craniofacial Research, National Institutes of Health, Bethesda, MD, USA.; 14 Laboratory of Infectious Diseases, National Institute of Allergy and Infectious Diseases, National Institutes of Health, Bethesda, MD, USA.

## Abstract

Interpretation of severe acute respiratory syndrome coronavirus 2 (SARS-CoV-2) serosurveillance studies is limited by poorly defined performance of antibody assays over time in individuals with different clinical presentations. We measured antibody responses in plasma samples from 128 individuals over 160 days using 14 assays. We found a consistent and strong effect of disease severity on antibody magnitude, driven by fever, cough, hospitalization, and oxygen requirement. Responses to spike protein versus nucleocapsid had consistently higher correlation with neutralization. Assays varied substantially in sensitivity during early convalescence and time to seroreversion. Variability was dramatic for individuals with mild infection, who had consistently lower antibody titers, with sensitivities at 6 months ranging from 33 to 98% for commercial assays. Thus, the ability to detect previous infection by SARS-CoV-2 is highly dependent on infection severity, timing, and the assay used. These findings have important implications for the design and interpretation of SARS-CoV-2 serosurveillance studies.

## Teaser

Antibody detection of prior SARS-CoV-2 infection depends on severity, assay, and timing with implications for serosurveillance.

## INTRODUCTION

Despite advances in severe acute respiratory syndrome coronavirus 2 (SARS-CoV-2) prevention and treatment, the novel coronavirus continues to infect individuals at an unprecedented rate. Because vaccination programs remain limited in scope, millions of individuals worldwide continue to rely on natural postinfection immunity for protection from reinfection. Serosurveillance studies measuring the prevalence of antibodies to SARS-CoV-2 have been and will continue to be a key means for estimating transmission over time and extrapolating potential levels of immunity in populations, although precise correlates of protection have yet to be established. However, limited available data on the sensitivity of antibody assays to detect prior infection—particularly in appropriately representative populations and over time—make it difficult to accurately interpret results from these studies (*1*). For these reasons, longitudinal characterization of antibody responses following SARS-CoV-2 infection with a range of clinical presentations is an important research gap and will be critical to interpreting seroepidemiological data and informing public health responses to the pandemic.

Infection with SARS-CoV-2 is associated with substantial variability in disease presentation, with severity ranging from asymptomatic infection to the need for high-level oxygen support and mechanical ventilation (*2*, *3*). There appear to be important relationships between the severity of illness and the magnitude and durability of the antibody response (*4*–*12*), but limited data are available evaluating the contributions of demographic factors and clinical features. Numerous platforms are available for the detection of antibody responses to SARS-CoV-2, which rely on different viral antigens and use different assay methods, and there is no guarantee that they will provide comparable data. With a few notable exceptions (*6*, *7*), most studies to date have produced antibody data from a single or limited number of platforms to evaluate antibody responses following infection (*8*–*10*, *13*–*15*). Comparisons across platforms and assay format differences (e.g., direct versus indirect detection), including the correlation between binding assays and neutralization capacity, have thus far been limited (*4*, *7*, *16*).

Here, we characterize the antibody responses to SARS-CoV-2 among a diverse cohort of individuals with documented infection, with a focus on investigating (i) the determinants of the magnitude and durability of humoral immune responses across a spectrum of disease severity; (ii) the relationship between antibody responses across a wide variety of binding assay platforms (13 total) and their correlation with neutralization capacity; and (iii) the implications of individual, temporal, and assay variability for serosurveillance. Our findings provide insight into the interpretation of antibody test results and have important implications for our understanding of humoral immunity to natural infection as well as for serosurveillance.

## RESULTS

### Participant demographics and characteristics

As shown in Table 1, the cohort of 128 participants had an average age of 48 years (range: 19 to 85 years) and was relatively balanced in terms of sex (45% female at birth), and 26% of participants self-identified as being of Latinx ethnicity, a group that has been identified to be at risk for coronavirus disease 2019 (COVID-19). Common medical comorbidities were hypertension (23%), lung disease (16%), and diabetes (13%). Notably, 18 individuals (14%) were living with HIV infection; 17 of 18 were on antiretroviral therapy. A total of 121 individuals (95%) reported symptoms during their COVID-19 illness, but only 31 (24%) required hospitalization. Among those who had been hospitalized, 84% required supplemental oxygen, 42% required intensive care unit (ICU) admission, and 13% required mechanical ventilation. A minority of individuals (*n* = 7, 5%) were asymptomatic.

**Table 1:** Demographic and clinical characteristics of the study participants. BMI, body mass index; ICU, intensive care unit.

**Characteristic**	***N* = 128 (%)**
**Age (years)**	47.8 (range: 19–85)
**Female sex at birth**	57 (44.5)
**Race**	
American Indian or Alaska Native	4 (3.1)
Asian	15 (11.7)
Black or African American	7 (5.5)
Native Hawaiian or Other Pacific Islander	3 (2.3)
White	81 (63.4)
Declined	20 (15.6)
**Latinx ethnicity**	33 (25.8)
**Medical comorbidities**	
Autoimmune disease	9 (7.0)
Active cancer	3 (2.3)
Diabetes	17 (13.3)
HIV	18 (14.1)
Heart disease	3 (2.3)
Hypertension	29 (22.7)
Lung disease	21 (16.4)
Kidney disease	2 (1.6)
Obesity (BMI ≥ 30)	38 (29.7)
**Clinical manifestations of COVID-19**	
**Asymptomatic**	7 (5.5)
**Symptomatic**	121 (94.5)
Fever	86 (70.5)
Chills	75 (61.5)
Fatigue	110 (90.2)
Cough	89 (73.0)
Shortness of breath	77 (63.1)
Rhinorrhea	58 (47.5)
Sore throat	56 (45.9)
Myalgias	84 (68.9)
Nausea	36 (29.5)
Vomiting	12 (9.8)
Diarrhea	50 (41.0)
Anosmia or dysgeusia	82 (67.2)
Headache	77 (63.1)
**Hospitalized**	31 (24.2)
Required supplemental oxygen	26 (83.9)
Required ICU admission	13 (41.9)
Required mechanical ventilation	4 (12.9)
**Numbers of symptoms reported**	
1–3	13 (10.2)
4–6	34 (26.6)
7–9	47 (36.7)
10–13	27 (21.1)
**Enrollment and follow-up**	
Baseline visit, days since onset (median)	63 (range: 22–157)
Follow-up time, days since onset (median)	110 (range: 22–157)
Time points contributed (median)	2 (range: 1–4)

The baseline visit for participants occurred at a median of 63 days (range: 22 to 157) after symptom onset (fig. S1). Participants contributed a median of 2 samples each (range: 1 to 4) and were followed up for a median of 110 days after symptom onset (range: 22 to 157). A total of 267 samples from the 128 enrolled participants were tested using at least 1 of the 14 assays evaluated; 171 samples from 88 individuals were tested using all 14 assays (Table 2 and fig. S2). Individuals with HIV were excluded from the Neut-Monogram as noted in Materials and Methods.

**Table 2:** Description of each assay. Unit abbreviations: S/C, sample result to calibrator result index; COI, cutoff index; AU/ml, arbitrary unit per milliliter; ID_50_, 50% inhibitory dilution; RLU, relative light unit; LU, light unit; conc, relative concentration. Antigen abbreviations: N, nucleocapsid; S, spike; RBD, receptor binding domain.

**Assay**	**Shorthand**	**Cutpoint (units)**	**Antigen**	**Sensitivity**	**Specificity**	**Analytical scale**
**Commercial**						
Abbott ARCHITECT SARS- CoV-2 IgG	N-Abbott	1.4 (S/C)	N	99.6	100	Natural
Roche Elecsys anti–SARS- CoV-2 total	N-Roche	1.0 (COI)	N	99.5	99.8	Log
Ortho Clinical Diagnostics VITROS anti–SARS-COV-2 total	S-Ortho Ig	1.0 (S/C)	S	100	100	Natural
Ortho Clinical Diagnostics VITROS Anti-SARS-CoV-2 IgG	S-Ortho IgG	1.0 (S/C)	S	90	100	Natural
DiaSorin LIAISON SARS-CoV- 2S1/S2 IgG	S-DiaSorin	15.0 (AU/ml)	S1/S2	97.6	99.3	Natural
Monogram PhenoSense Assay	Neut-Monogram	40.0* (ID_50_)	S	100	98.8	Log
**Research use**						
Split luciferase (total Ig)	RBD-Split Luc	45.9 (RLU)	RBD	89	100	Log
Split luciferase (total Ig)	N-Split Luc	83.1 (RLU)	N	98	99	Log
Luciferase Immunoprecipitation System Assay (total Ig)	RBD-LIPS	52,000 (LU)	RBD	94	100	Log
Luciferase Immunoprecipitation System Assay (total Ig)	N-LIPS	125,000 (LU)	N	100	100	Log
Luminex (IgG)	S-Lum	0.0426 (conc)	S	91.5	100	Log
Luminex (IgG)	RBD-Lum	0.0396 (conc)	RBD	91.9	100	Log
Luminex (IgG)	N(full)-Lum	0.02473 (conc)	N	85	100	Log
Luminex (IgG)	N(frag)-Lum	0.02684 (conc)	N	65.8	100	Log

*The cutpoint for Neut-Monogram is the lower limit of detection for the assay.

### Substantial heterogeneity in antibody responses across individuals and assays

**Fig. 1:**
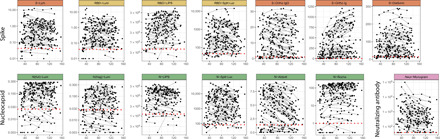
Longitudinal antibody kinetics. Time since symptom onset is shown on the *x* axis versus the measured antibody response for each assay. For asymptomatic individuals, the time since the first positive polymerase chain reaction (PCR) test was used. Black points indicate individual time points, and longitudinal samples are connected with gray lines. *y* axes are transformed as indicated in Table 2. Assay units are as follows: S-Lum (conc, relative concentration), RBD-Lum (conc, relative concentration), RBD-LIPS (LU, light unit), RBD-Split Luc (RLU, relative light unit), S-Ortho IgG (S/C, sample result to calibrator result index), S-Ortho Ig (S/C, sample result to calibrator result index), S-DiaSorin (AU/ml, arbitrary unit per milliliter), N(full)-Lum (conc, relative concentration), N(frag)-Lum (conc, relative concentration), N-LIPS (LU, light unit), N-Split Luc (RLU, relative light unit), N-Abbott (S/C, sample result to calibrator result index), N-Roche (COI, cutoff index), and Neut-Monogram (ID_50_, 50% inhibitory dilution). Red dotted lines indicate cutoff values for positivity, as indicated in Table 2.

We observed substantial heterogeneity in measured antibody responses in individuals at baseline and throughout follow-up across all assays (Fig. 1; raw data are available in tables S1 and S2). We observed variable trajectories of antibody responses between assays, with some [N-Abbott, N-Split Luc, S-Ortho immunoglobulin G (IgG), and Neut-Monogram] showing a clear decrease over time, other assays (S-Ortho Ig and N-Roche) showing a clear increase, and the remainder with more stable values (Fig. 1 and table S3). When comparing antibody levels between individuals, responses were very heterogeneous, with some individuals mounting strong responses for all assays and others with weak responses even at the initial visit (below the positivity cutoff for some assays).

### Strong correlation between binding and neutralization assays

**Fig. 2:**
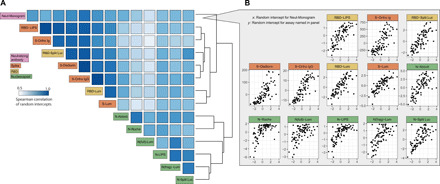
Correlation of responses between assays. (**A**) Spearman correlation of random intercepts derived from a mixed-effects model, representing responses at 21 days after symptom onset for each individual from the longitudinal data. Assays are sorted by hierarchical clustering using average distance clustering. Darker blue indicates higher correlation; colored label box indicates antigen for each binding assay and the neutralizing assay. (**B**) Pairwise scatterplots showing the random intercepts for the neutralizing assay (*x* axis) versus the random intercepts for each of the other assays (*y* axis). Assay units are indicated in Table 2.

We observed high levels of correlation between estimated antibody levels at 21 days after symptom onset (random intercept) for all assays, with Spearman correlations ranging between 0.55 and 0.96 (Fig. 2A and fig. S3). Rank correlations were consistently higher between binding assays using the same antigenic target [spike (S)/receptor binding domain (RBD) versus nucleocapsid (N)] than between those using different targets, despite the variety in platforms used and the measurement of responses to both targets on some platforms [luciferase immunoprecipitation systems (LIPS), Luminex, split luciferase]. Titers of neutralizing antibodies correlated well with all binding assays (range: 0.60 to 0.88) and correlated most highly with responses to the S protein (range: 0.76 to 0.88), as might be expected given the expression of S protein on the pseudovirus used in the neutralization assay (Fig. 2B and fig. S3). We found no substantive differences in correlations between binding and neutralization assays at time points before versus after 90 days, suggesting that these relationships did not appreciably change over the duration of observed follow-up (table S4).

### Disease severity is strongly associated with the magnitude of antibody responses

**Fig. 3:**
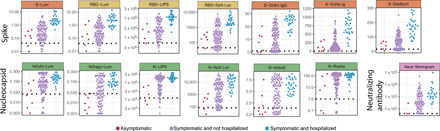
Severity-stratified antibody response at baseline. Swarm plot of antibody response at the baseline visit for each study participant by assay, stratified into individuals who experienced no symptoms, individuals who experienced symptoms but were not hospitalized, and those who experienced symptoms and were hospitalized. *y* axes are transformed as indicated in Table 2.

Baseline antibody responses for each study participant showed remarkably consistent patterns across all assays when stratified by severity class, with asymptomatic individuals having the lowest responses, hospitalized individuals having the highest, and symptomatic but not hospitalized individuals having intermediate responses (Fig. 3). While the number of asymptomatic individuals was small, responses were substantially lower in these individuals than those who were symptomatic but not hospitalized for multiple assays; hospitalized individuals had significantly higher responses than both other groups for all assays with the notable exception of the neutralization assay (table S5). Despite these consistent patterns, there was still substantial variation in the magnitude of responses between participants within each severity category. Notably, age, sex, HIV status, and Latinx ethnicity showed little association with antibody responses after adjusting the analysis for hospitalization (table S6).

### Need for hospitalization, cough, and fever are key predictors of antibody responses

**Fig. 4:**
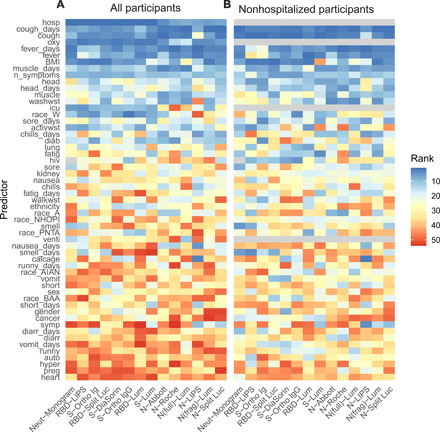
Clinical predictors of antibody responses. Rank of variable importance (1 = highest rank; 50 = lowest rank) in a random forest classifier of top half versus bottom half of responders for each assay, based on random intercepts, including (**A**) all individuals (*n* = 128) and (**B**) only individuals who were not hospitalized (*n* = 97), as hospitalization is a strong predictor of antibody response. Variable importance was determined as the reduction in classification error averaged across 10 runs of the algorithm. Variables only relevant to hospitalized individuals (i.e., whether the individual was hospitalized, whether oxygen was required, whether the individual was in the ICU, and whether the individual required a ventilator) were omitted from the classifier in (B) and shown in gray. In addition, HIV status is excluded as a predictor for the Neut-Monogram assay for reasons described in the main text. The dependent variable (individual-level random intercepts derived from a mixed-effects model) is dichotomized into “high” and “low,” determined by the random intercept being in the upper or lower half of all random intercepts for that assay, respectively. Full labels of the predictor variables are provided in table S10.

We next examined which of the 50 individual demographic and clinical variables were the strongest predictors of the magnitude of the antibody response (top versus bottom half of responders for each assay; fig. S4) using a random forest algorithm. Among the entire cohort (*n* = 128), the presence and duration of cough and fever and the need for hospitalization and supplemental oxygen during the initial illness were the most important predictors of the antibody response (Fig. 4A). The ranks of their importance varied subtly but were largely consistent across the 14 assays evaluated, and random forest models including only these six variables were able to predict high versus low magnitude of response on each assay with reasonably high accuracy [areas under the curve (AUCs) ranging from 0.74 to 0.86; table S7]. Among those individuals who were not hospitalized (*n* = 96), the presence and duration of fever and cough remained the most important predictors of a high antibody response (Fig. 4B). These four variables alone were predictive of high versus low responses within this subset of individuals with modest accuracy (all except RBD-LIPS with AUCs above 0.6).

### Time to seroreversion varies considerably across platforms and by infection severity

**Fig. 5:**
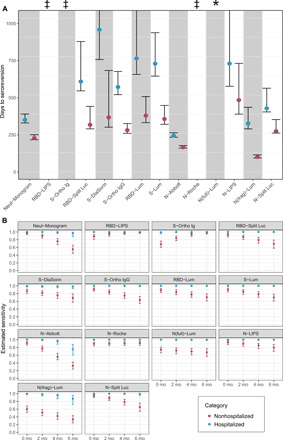
Estimated time to seroreversion and assay sensitivity by time and hospitalization status. (**A**) Mean time to seroreversion for individuals tested on each assay, stratified by hospitalization status, with 95% confidence intervals derived from bootstrapping. The symbol “‡” indicates increasing antibody responses over time (95% confidence interval for time to seroreversion was negative and did not cross 0), and the symbol “*” indicates antibody responses for which 95% confidence interval of time to seroreversion crossed 0. (**B**) Estimated sensitivity of each assay (showing posterior median estimates and 95% credible intervals), stratified by hospitalization status at 2-month intervals, from 0 to 6 months after seroconversion. Seroconversion was assumed to occur (if at all) 21 days after symptom onset (if symptomatic) or 21 days after positive PCR test (if asymptomatic).

Using the mixed-effect model described above, we estimated the expected time to seroreversion (when antibodies would become undetectable for an average individual) for each platform, assuming that antibody responses changed linearly over time. We estimated separate times to seroreversion for hospitalized and nonhospitalized individuals, because hospitalization status was a strong predictor of baseline antibody status. Estimated time to seroreversion was substantially shorter for nonhospitalized versus hospitalized individuals for all assays, consistent with the lower initial antibody titers in those individuals. For those assays where antibody levels decreased over time, we also observed marked variation in times to seroreversion between assays, ranging from 96 days for N(frag)-Lum to 925 days for S-DiaSorin; the estimated time to seroreversion is infinity for RBD-LIPS, S-Ortho Ig, and N-Roche, which exhibited increasing mean antibody responses over time in this dataset (Fig. 5A and table S3).

### Sensitivity of assays to detect prior infection varies as a function of time and infection severity

We next assessed how sensitivity of each platform varied as a function of time and disease severity. Because the sample size of asymptomatic individuals was small, they were grouped with other nonhospitalized individuals for this analysis (fig. S5). We found considerable heterogeneity in sensitivity between assays and as a function of illness severity and time since infection. Across all 14 assays, sensitivity at each time point was higher in the hospitalized subset of the cohort than in the nonhospitalized subset, the latter group representing the majority of infections in the general population (Fig. 5B and table S8). The magnitude of this difference varied between assays and also over time and was often considerable (fig. S6). Estimated sensitivity declined over time for 11 of the 14 assays but increased for RBD-LIPS, S-Ortho Ig, and N-Roche, consistent with the observed increase in magnitude of response over time for these assays. Overall, RBD-LIPS showed the most consistently high sensitivity over time and the smallest difference between hospitalized and nonhospitalized individuals, ranging from 88% (95% credible interval: 81 to 94%) at month 0 to 99% (95% credible interval: 96 to 100%) at month 6 in nonhospitalized individuals. Of the remaining research-use assays, N-LIPS, RBD-Split Luc and N-Split Luc, and RBD-Lum and S-Lum showed a similar pattern, with high sensitivity initially followed by a decline in sensitivity among nonhospitalized individuals over time; N(full)-Lum and N(frag)-Lum had consistently poor sensitivity for nonhospitalized individuals. All commercial assays performed similarly well during early convalescence, albeit with lower sensitivity for nonhospitalized individuals for S-DiaSorin and S-Ortho Ig. N-Abbott showed the greatest decline in sensitivity with time and the greatest difference between hospitalized and nonhospitalized participants, with sensitivity varying from 100% (95% credible interval: 100 to 100%) in hospitalized individuals soon after infection to 33% (95% credible interval: 24 to 42%) in nonhospitalized individuals at 6 months. Of note, neutralization titers remained detectable for nearly all hospitalized individuals up to 6 months but were estimated to become undetectable on this assay for nearly half of nonhospitalized individuals by this time point.

### Varying assay sensitivity affects interpretation of individual antibody test results

**Fig. 6:**
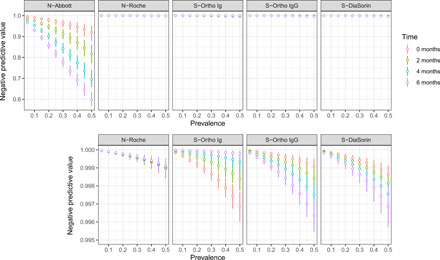
Negative predictive values of the commercial assays. Negative predictive values shown are based on the estimated assay sensitivities for nonhospitalized individuals in Fig. 5B, for a range of prevalence between 5 and 50% (*x* axis). Bottom panels show the same data with a smaller range in the *y* axis to visualize small differences.

Last, negative predictive values were calculated for the commercial assays to illustrate the potential effects of these changes in sensitivity if the assays were used to ascertain prior infection in individuals (assuming values of specificity as reported by the manufacturers). As expected, negative predictive values decreased with increasing prevalence, except for S-Ortho Ig and N-Roche (Fig. 6).

## DISCUSSION

This study documents the large heterogeneity in longitudinal antibody responses to SARS-CoV-2 across a large number of commercial and research assays in a diverse cohort of individuals. Measured responses in all binding assays correlated well with each other and, particularly for those measuring responses to spike protein, with pseudovirus neutralization. For all assays, we found a consistent, strong, and dose-dependent effect of disease severity on antibody magnitude. Despite these similarities, assays performed quite differently in terms of sensitivity to detect prior infection and in the durability of measured responses, leading to large discrepancies in sensitivity between assays in the months following infection. Thus, the ability to detect previous infection by SARS-CoV-2 using an antibody test is highly dependent on the severity of the initial infection, when the sample is obtained relative to infection, and the assay used.

Prior work has shown that antibody responses in individuals with symptomatic COVID-19 have, in some cases, been associated with disease severity (*4*–*12*). We observed significant variability in antibody responses between study participants, which was largely explained by the self-reported symptom constellation and the severity of the acute illness. A few simple variables consistently predicted the magnitude of the antibody responses across multiple assay platforms and antigen targets; these symptoms (e.g., fever and cough) are similar to those recently described in a population-based Icelandic cohort (*6*). In contrast to that cohort, characteristics like age and sex were not predictive of these responses, after accounting for disease severity, although some univariate associations with antibody responses were significant. This suggests that disease severity may underlie some of these apparent relationships or could alternatively be explained by nonrepresentative enrollment in the Long-term Impact of Infection with Novel Coronavirus (LIINC) study.

We also observed substantial heterogeneity between assays in terms of overall sensitivity, particularly over time. These findings build upon prior work showing differences in sensitivity during early convalescence (*7*, *10*). This finding may also provide insight into apparent discrepancies in previous studies that have reported different durabilities of antibody responses (*13*, *17*). Differences in performance were particularly pronounced among nonhospitalized individuals, who have lower antibody responses and comprise the majority of those infected with SARS-CoV-2. Notably, antibody responses in such individuals are expected to be reliably detectable over 6 months in only two of the commercial binding assays tested—S-Ortho Ig and N-Roche—which were the only ones to use direct detection format of antibodies (to different viral targets). These two assays, along with a research-use assay using direct detection (RBD-LIPS), were the only ones to demonstrate increasing rather than decreasing antibody signal over time, possibly due to continued affinity maturation of the antibody response (*18*) playing a larger role in detection with this format. This is in contrast to the indirect format assays and neutralization assay, all of which demonstrated waning over time. Another possible explanation for discrepancies between assays is that only assays with the highest signal-to-noise ratio are able to consistently detect antibodies above background in those with the lowest titers, i.e., those multiple months out from mild infections. This variation in sensitivity is relevant for several reasons. First, it provides further evidence that use-cases need to be considered when evaluating performance of antibody tests. While all evaluated assays had near-perfect sensitivity for detecting antibody responses among hospitalized individuals and therefore could be useful as an indirect diagnostic tool in that setting, their sensitivity to detect responses in the general population, where most infections are mild, is much lower and quite variable (*1*). Second, it implies that using assays with decreasing sensitivity over time for population seroprevalence studies will underestimate the true proportion of previously infected individuals and that this underestimation will be more substantial as the amount of time that has passed since infection increases. Third, it shows dramatic differences between the sensitivity reported by test manufacturers, often limited to validation sample sets from hospitalized and/or recently infected individuals that were readily available early in the pandemic, and the expected sensitivity in the general population. Because of this, our study provides information that could be useful for assay selection when planning future serosurveys and could help correct the interpretation of large-scale population-based seroprevalence studies that have used some of these assays. Last, individual patients or providers using these assays to assess the presence or absence of prior infection and/or immune status should take these considerations into account, given the poor negative predictive value of some tests.

This analysis has several notable strengths, including the utilization of a broad array of antibody platforms at multiple time points in a diverse cohort of individuals with varying degrees of illness severity and rich clinical phenotyping. However, there are several limitations. The cohort was not population-based and therefore not truly representative of all individuals with SARS-CoV-2 infection. Despite this, we endeavored to recruit a cohort that spanned the spectrum of SARS-CoV-2 infection. Second, this analysis included only a small number of asymptomatic individuals, which are likely to differ from symptomatic patients in terms of initial and possibly longitudinal responses based on our limited data and prior results (*11*). Additional studies in larger numbers of asymptomatic and paucisymptomatic individuals will be necessary to inform accurate interpretation of serosurvey results. Third, the current analysis is limited to samples obtained up to 4 months after infection. Data from longer follow-up times will be very useful to estimate the longer-term kinetics of antibody responses in all platforms with more certainty. The assumption of linearity, while empirically appropriate on the time scales of data that we have here, may not be accurate for extrapolation into the distant future as antibody responses often follow more complex dynamics of boosting and waning over time (*19*). Last, it is important to recognize that assays optimally suited for serosurveillance may not be equally suitable for other use-cases, such as identifying recent infection, detecting reinfection, determining protective capacity, or determining potency of COVID-19 convalescent plasma (*20*). Evaluating the performance of assays for each of these use-cases will require different study designs and sample sets.

As SARS-CoV-2 vaccination becomes a reality, many serosurveillance efforts will need to increasingly rely on assays that can distinguish vaccination from natural infection, especially when it is not possible to obtain additional information on the vaccination status of participants. Currently distributed vaccines in the United States are based on S protein; thus, responses to S-based assays will likely reflect a combination of natural infection and vaccination, whereas assays based on N will reflect only natural infection. While several of the N-based assays we evaluated performed well, one of the two commercially available N-based assays demonstrated substantial waning of sensitivity over time, which will affect estimates of the seroprevalence of natural infection but could be a more useful indicator of recent infection along with other potential markers such as IgM. Further studies will be needed to characterize the performance of these and other assays for serosurveillance in the presence of vaccine-induced immunity.

In this study, we demonstrated substantial differences in the detectability of antibody responses to SARS-CoV-2 related to illness severity, time since infection, and assay platform. These results will be important in choosing and interpreting serologic assays for evaluating infection and immunity in population surveillance studies.

## MATERIALS AND METHODS

### Study cohort

Participants were volunteers in the University of California, San Francisco–based LIINC natural history study (NCT04362150). All volunteers signed informed consents for the study. LIINC is an observational cohort that enrolls individuals with SARS-CoV-2 infection documented by clinical nucleic acid amplification testing who have recovered from the acute phase of infection. Volunteers are recruited by clinician or self-referral. They are eligible to enroll between 14 and 90 days after onset of COVID-19 symptoms and are offered monthly visits until 4 months after illness onset; they are then seen every 4 months thereafter.

Clinical data from the initial LIINC study visit were used for this analysis. At this visit, LIINC participants underwent a detailed clinical interview conducted by a study physician or research coordinator using a standardized data collection instrument. Demographic data collected included age, sex, gender, race, ethnicity, education level, income level, and housing status. Data related to SARS-CoV-2 infection included the date and circumstances of diagnosis, illness, and treatment history. Each participant was asked to estimate the date of symptom onset in relation to the timing of their first SARS-CoV-2 nucleic acid amplification test result. Participants were questioned regarding the presence, duration (in days), and current status of a list of COVID-19 symptoms and additional somatic symptoms derived from the Patient Health Questionnaire (*21*), as well as measures of quality of life derived from the EQ-5D-5L Instrument (*22*). We determined from medical records whether each individual was hospitalized (defined as spending >24 hours in the emergency department or hospital) and whether they required supplemental oxygen, admission to an ICU, or mechanical ventilation. Past medical history was ascertained, and concomitant medications were recorded.

At each visit, blood was collected by venipuncture. Serum and plasma were isolated via centrifugation of nonanticoagulated and heparinized blood, respectively, and stored at −80°C. For the current analyses, we included 128 participants who were enrolled between April and July 2020 and who had at least one measurement on a binding assay or neutralization platform.

### Antibody assays

Table 2 describes each of the assays performed as part of this analysis, including their sensitivity and specificity (as reported by the manufacturers for commercial assays or by validation testing for noncommercial research assays). Commercially available platforms included the Abbott ARCHITECT (IgG), Ortho Clinical Diagnostics VITROS (IgG and total Ig), DiaSorin LIAISON (IgG), Roche Elecsys (total Ig), and Monogram PhenoSense (neutralizing antibodies) assays according to the manufacturer’s specifications. Individuals living with HIV infection were excluded from analyses involving the Monogram PhenoSense assay, which uses an HIV backbone for the pseudovirus. Noncommercial research use assays included the LIPS assay (total Ig) targeting the N protein and the RBD of the S protein performed in the Burbelo laboratory (additional raw data on responses to full S protein, highly correlated with RBD responses, are included in tables S1 and S2) (*23*); the split luciferase assay (total Ig) targeting N and S performed in the Wells laboratory (*24*); and the Luminex assay (IgG) targeting N (one full-length and one fragment), S, and RBD performed in the Greenhouse laboratory.

For the research use Luminex assay, we used a published protocol with modifications (*25*). Plasma samples were diluted to 1:100 in blocking buffer A (1× phosphate-buffered saline, 0.05% Tween, 0.5% bovine serum albumin, and 0.02% sodium azide). Antigens were produced using previously described constructs (*26*, *27*). Antigen concentrations used for COOH-bead coupling were as follows: S, 4 μg/ml; RBD, 2 μg/ml; and N, 3 μg/ml. Concentration values were calculated from the Luminex median fluorescent intensity (MFI) using a plate-specific standard curve consisting of serial dilutions of a pool of positive control samples. Any samples with MFIs above the linear range of the standard curve were serially diluted and rerun until values fell within range to obtain a relative concentration. A cutoff for positivity was established for each antigen above the maximum concentration value observed across 114 prepandemic SARS-CoV-2 negative control samples tested on the platform.

### Statistical analyses

#### Comparing individuals across assays and estimating time to seroreversion

For each assay, we fit a linear mixed-effects model that included a patient-specific random intercept. Given the longitudinal nature of our dataset, we fit mixed-effects models to explicitly account for the repeated measurement of individuals over time. We log-transformed the response variable for a subset of the assays based on assessment of their correlations with log-transformed neutralization titers (Table 2). For observation *h* for individual *i*, we modeled their antibody response *Y_hi_* on each assay as follows

$$Y_{\mathrm{hi}}=\beta_{s}+\lambda {\text{Time}}_{\mathrm{hi}}+u_{0i}+e_{\mathrm{hi}}$$ (1)

In Eq. 1, β*_s_* represents the overall mean for severity class *s*, where *s* was dichotomized into whether an individual was hospitalized or not hospitalized; λ represents the fixed effect of *Time_hi_*, where *Time_hi_* is data on the time since symptom onset (if symptomatic) or since positive polymerase chain reaction (PCR) test (if asymptomatic). In addition, *u*_0*i*_ represents an individual-level random effect that is normally distributed with a mean of 0 and an SD of τ, and *e_hi_* represents the residual error that is normally distributed with a mean of 0 and an SD of σ. We also considered models that included additional fixed effects for covariates such as age, ethnicity, sex, and HIV status (table S6). We did not find consistent differences in the slope of the antibody responses (λ) by hospitalization status across the majority of the assays used here; therefore, we used a single slope for each assay throughout (table S9).

Because the timing of the baseline visit was variable between individuals, to directly compare the magnitude of measured responses for individuals on each assay, we used the mixed-effects model to estimate the antibody response that each person would have at 21 days after symptom onset (random intercept); we chose this value as the time origin to reflect the time from symptom onset to seroconversion (*28*). For comparability across assays, we also used the model estimates to calculate the mean time to seroreversion *T* for severity class *s* on each assay, given the cutoff value for positivity (Table 2), as follows

$$T_{s}=(\mathrm{cutoff}-\beta_{s})/\lambda$$ (2)

We performed bootstrapping to obtain 95% confidence intervals of *T_s_* for each of the 14 assays. We used the time to seroreversion as the outcome here rather than alternative quantities such as the half-life, as the serologic responses obtained here did not all necessarily represent direct measurements of antibody titers. The calculation of time to seroreversion assumes that the slope, λ, is maintained over time. These models were fit using the lme4 package using the R statistical software (www.R-project.org/).

#### Random forest modeling of demographic/clinical predictors and antibody responses

For each assay, we used random forests to model antibody responses based on 50 demographic and clinical predictors (table S10). We dichotomized the antibody response for each individual on each assay based on whether their estimated random intercept was in the top half or the bottom half of all fitted random intercepts on that assay. We first fit models to these dichotomized antibody responses using all available predictors; subsequently, we fit models to these dichotomized antibody responses on a down-selected set of predictors selected based on variable importance (i.e., mean decrease in accuracy). We quantified prediction accuracy using the out-of-bag error rate and the AUC. These models were built using the randomForest package using the R statistical software (version 3.5.3).

#### Estimating time-varying assay sensitivity

For each assay, we fit an extension of the linear mixed-effects models described in Eq. 1 above in a Bayesian hierarchical modeling framework, where we allowed the SD of the random intercept (τ) to be severity specific (now referred to as τ*_s_*). For parsimony, we assumed the slopes, λ, to again be shared across all individuals as described above. To estimate changes in assay sensitivity over time, we simulated population distributions of Ŷ by iteratively sampling values from each of the posterior distributions of β*_s_*, λ, τ*_s_*, and σ. For each sampled value of τ*_s_* and σ, we then sampled draws of *u*_0*i*_ and *e_hi_* and determined the assay sensitivity at various time points (0, 60, 120, and 180 days after seroconversion) as the proportion of overall draws where Ŷ was above the cutoff value for positivity. We ran four Markov chain Monte Carlo chains of length 2000 each using the Stan programming language (https://mc-stan.org/) and assessed convergence using the Gelman-Rubin Rhat statistic. We used uninformative priors for all parameters and hyperparameters. Data and code to reproduce all analyses are available at https://github.com/EPPIcenter/liinc-Ab-dynamics.

### Ethical considerations

All participants signed a written informed consent form. The study was approved by the University of California, San Francisco Institutional Review Board.

## SUPPLEMENTARY MATERIALS

Supplementary material for this article is available at http://advances.sciencemag.org/cgi/content/full/7/31/eabh3409/DC1

## Appendix

View/request a protocol for this paper from *Bio-protocol*.
